# PROFESSOR JOAQUIM JOSÉ GAMA-RODRIGUES. FORMER PRESIDENT OF THE BRAZILIAN COLLEGE OF DIGESTIVE SURGERY

**DOI:** 10.1590/0102-672020240004e1797

**Published:** 2024-04-19

**Affiliations:** 1 São Leopoldo Mandic, Faculty of Medicine, Campinas (SP), Brazil;; 2 Santa Casa de São Paulo, Hospital and Medical School, Bariatric and Gastroesophageal Unit, São Paulo (SP), Brazil.

**Figure:**
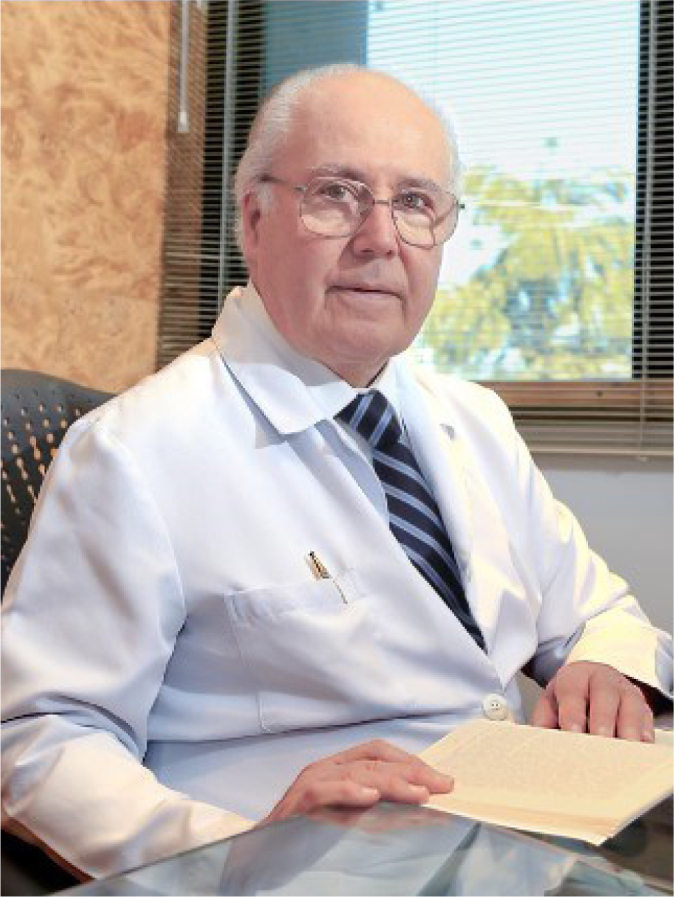


Professor Joaquim José Gama-Rodrigues was born in Cruzeiro, state of São Paulo, Brazil, on December 2, 1935. Following his family's example and vocation, he pursued education in public schools, initially in Guaratinguetá, near his hometown, and later attended *Colégio Estadual Presidente Roosevelt* in São Paulo city.

He successfully passed the entrance exam and immediately gained admission to the Faculty of Medicine of *Universidade de São Paulo* (FMUSP) in 1954, ranking 38^th^ among his peers. He never left until compulsory retirement, at age 70, in 2005.

His vocation has always been the incessant pursuit for knowledge, following the example set by his mentors, to increasingly expand his expertise. This drive led him to undertake scientific initiation at the Surgical Technique Department, where his efforts culminated in a study published in an indexed journal while he was still a student.

He consistently prioritized sharing knowledge with his peers, leading him to assume roles such as the speaker of his class and student representative at the National Union of Students (UNE) and State Union of Students (UEE) on numerous occasions. This associative activity persisted throughout his academic journey, initially as a representative of physicians and later as a representative of full professors and associates of FMUSP.

In 1960, he graduated and achieved 1^st^ place in the Residency in General Surgery exam, at the *Hospital das Clínicas* of FMUSP. His excellence was further recognized when he was elected chief resident by his peers. At that time, he held a seat on the Administrative Committee, and actively contributed to the laying of the "foundation stone" for the building that accommodates hospital residents.

In this nurturing environment, he found himself drawn to morphological subjects, an area in which the character and intellectual discipline of Renato Locchi's inspiring personality stood out. His interest in surgery was quickly piqued, at which point he received invaluable encouragement and support from extraordinary mentors at the onset of his journey. The esteemed names of Alípio Corrêa Netto and Arrigo Raia deserve mention. As a recent graduate, he also benefited from the valuable guidance of Antonio Barros de Ulhôa Cintra and Carlos da Silva Lacaz, among many other luminaries in the field. Such an exceptional experience prompted him to contemplate the need to always be prepared to fulfill the institutional requirements that evolve within work communities over time. He realized that continuous personal improvement was an inevitable obligation in this pursuit.

He obtained doctorate in medicine with the thesis entitled "Motor changes of the esophagus in patients with esophageal varices caused by schistosomiasic portal hypertension" in 1972^1^; the title of Associate Professor with the thesis entitled "Sliding Hiatal Hernia. Esophageal Fundogastropexy Associated with Hiatoplasty, Clinical, Morphological and Functional Assessment" in 1974^2^; and the title of Full Professor of Surgery in the Department of Gastroenterology of FMUSP, in 2002, through a public tender.

He held the position of Head of the Department of Gastroenterology and Surgery at FMUSP and Head of the Stomach and Small Intestine Surgery Service at the *Hospital das Clínicas* of the Universidade de São Paulo. Additionally, he was a full member of the Board of Directors of USP from 1990 until 2005.

In his associative endeavors, he served as a founding member and president of the Brazilian College of Digestive Surgery, 2001–2002. He was also the creator and founding member, along with several colleagues, of the Brazilian Gastric Cancer Association, for which he was elected as the association's first president, from 1999 to 2001, and again for the 2004–2006 biennium. His contributions led to the presidency of the International Gastric Cancer Association (IGCA) from 2007 to 2009. Furthermore, he served as the director of the Angelita and Joaquim Gama Institute for Research and Teaching in the Digestive System and is a founding member of the Brazilian Association for the Prevention of Bowel Cancer (ABRAPRECI) since 2004.

He is an honorary member of the American College of Surgeons, a title bestowed upon him in 2006, as well as an honorary member of the Brazilian College of Surgeons, awarded in 2018.

He participated in the organization of numerous medical congresses, notably serving as the president of the 7^th^ World Gastric Cancer Congress held in São Paulo, in May 2007. In addition, he is president of the Organizing Committee of the International Rectal Cancer Forum (*Fórum Internacional de Câncer do Reto* – FICARE), a biennial event held in São Paulo since 2007. He has made significant contributions through important publications on this topic^3,4^.

He has authored 251 scientific articles indexed in PubMed and boasts an impressive H-index of 54. He has held various positions in esteemed scientific journals, including member of the Editorial Committee of the World Journal of Surgery, Hepatogastroenterology, *Acta Cirúrgica Brasileira*, *Arquivos Brasileiros de Cirurgia Digestiva*, *Arquivos de Gastroenterologia*, and of Gastric Cancer; the first being based in the USA, the last at IGCA in Tokyo, and the others in São Paulo.

It is noteworthy to mention that Professor José Joaquim Gama-Rodrigues was a mentor and motivator for doctors, disciples, and researchers alike. He actively fostered opportunities and supported the career development of his peers, colleagues, and residents, participating in the creation of the Endoscopy Service at the *Hospital das Clínicas* of FMUSP. As director of the Stomach and Small Intestine Service, he headed the "GENOMA" project, a groundbreaking initiative aimed at advancing the study of Gastric Cancer. This project not only modernized and standardized gastric cancer surgery in Brazil and Latin America but also facilitated international collaborations, particularly with Japan and other international centers^5–7^. As a result of his efforts, this activity allowed him to bring the 7^th^ World Gastric Cancer Congress to our country in 2007, laying the foundation for its return in 2015.

In collaboration with Prof. Arrigo Raia, Prof. Angelita Habr-Gama, and Prof. Henrique Walter Pinotti, he played a role in the inception of the renowned GASTRÃO, the foremost Brazilian event in the realm of Digestive System Surgery and Gastroenterology, which has recently celebrated 50 years of continuous operation.

Building upon this foundation, in 1988, along with Prof. Henrique Walter Pinotti and other collaborators, he created the Brazilian College of Digestive Surgery. Six years later, in 1994, their efforts culminated in the formation of the medical specialty Digestive System Surgery. His performance was fundamental at the *Conselho Federal de Medicina* plenary session, for the acceptance and implementation of that specialty which has since become a cornerstone of medical practice in Brazil.

The Professor was the recipient of numerous accolades and distinctions, renowned for his excellence as a surgical educator and researcher. He was bestowed the title of Honored Professor by three graduating classes at FMUSP. He received honors from the Lusíada Academy of Sciences, Letters and Arts of São Paulo, where he was later appointed as an honorary member. In 2010, he was honored with the Order of Merit of Infante Dom Henrique and became an esteemed honorary member of Casa de Portugal in São Paulo.

Professor Joaquim José Gama-Rodrigues epitomizes the essence of a true **PROFESSOR**, embodying the qualities of a compassionate "human being," "doctor," "surgeon," and "teacher," leaving behind a rich cultural and humanistic legacy that serves as a beacon of guidance for generations to come.
